# Successful treatment of a unique case of congophilic fibrillary glomerulonephritis: A case report

**DOI:** 10.1097/MD.0000000000021101

**Published:** 2020-07-10

**Authors:** Pulkit Gandhi, Jie Tang

**Affiliations:** Division of Kidney Diseases and Hypertension, Alpert Medical School of Brown University, Providence, RI, United States.

**Keywords:** amyloidosis, Congo red stain, fibrillary glomerulonephritis

## Abstract

**Introduction:**

Amyloidosis and fibrillary glomerulonephritis (FGN) share similar electron microscopic signatures including random arrangement of fibrils. However, distinction between the 2 can often be made using Congo Red staining.

**Patient concerns:**

Here we describe a unique case of FGN, which stained positive for Congo Red, as well as DnaJ heat shock protein family (Hsp40) member B9 which is more specific for FGN. The patient presented with acute kidney injury and severe proteinuria.

**Diagnosis:**

Congophilic FGN.

**Interventions:**

Six-month course of mycophenolate mofetil and prednisone.

**Outcomes:**

complete resolution of acute kidney injury and proteinuria

**Take home lessons:**

To our knowledge, this is the first reported case of successful treatment of this rare condition using mycophenolate mofetil and prednisone.

**Key Points**

1. The final diagnosis of congophilic FGN was based on the pathological findings, as well as positive Congo Red & DNAJB9 stains.
2. Congo red positivity can rarely be seen in FGN in addition to amyloidosis. In such cases, DNAJB9 stain should be used to identify FGN.
3. A treatment regimen consists of MMF and prednisone may be considered in congophilic FGN.

## 1 Introduction

Glomerular diseases can be associated with fibrillar deposits in the glomerular basement membrane and mesangium. Congo Red staining has traditionally been used to distinguish between fibrillary glomerulonephritis (FGN) which is typically Congo Red negative, and amyloidosis which is Congo Red positive.^[1]^

FGN is a rare glomerular disease, seen in approximately 1% of native kidney biopsies.^[2]^ Most cases are idiopathic, although some are thought to be secondary to hepatitis C infection, paraproteinemia, or autoimmune diseases. The diagnosis is based on the typical morphological features seen on electron microscopy (EM). Patients usually present with renal insufficiency, hematuria and proteinuria, which can be in nephrotic range.^[3]^ On EM, fibril deposits can be seen either in mesangium, glomerular basement membrane, or both. Fibrils can vary in size, usually ranging between 16 to 24 nm, which is larger than those described in amyloidosis (4–11 nm).^[2]^ Under immunofluorescence, it can stain positive for Immunoglobulin G (IgG), complement component 3 (C3), Kappa And Lambda light chains.^[3]^ Certain IgG subclasses, such as IgG4, are seen more often than others.^[2]^ In general, FGN has a poor prognosis despite therapeutic interventions. Existing evidence showed that 43% of patients had persistent renal dysfunction, and 44% progressed to end-stage renal disease (ESRD) over an average of 4.3 years of follow-up.^[4]^

Here, we describe a rare case of FGN, which stained positive for Congo Red and DnaJ heat shock protein family (Hsp40) member B9 (DNAJB9). The patient responded well to a 6-month combined regimen of mycophenolate mofetil (MMF) and prednisone with complete remission.

## 2 Case presentation

A 58–year-old Caucasian male with medical history of hepatitis C status post treatment with Harvoni in 2015 with negative viral PCR in September 2016, well controlled type 2 diabetes mellitus (HbA1c 5.2%), well controlled hypertension (blood pressure 130's/50's), chronic kidney disease stage III with a baseline creatinine of 1.4 mg/dL and random urine albumin-to-creatinine ratio of 627 to 877 mg/g, presented as an outpatient consult for rising serum creatinine. The serum creatinine rose to 3.2 mg/dL over a period of 3 months. His 24 hour urine showed significant proteinuria, measured at 2682 mg. He did not report family history of kidney problems. Social history was unrevealing. He did not present with any systemic signs or symptoms. Systolic blood pressure ranged between 140 to 160's while in clinic. Physical examination did not reveal any pulmonary or cardiovascular abnormalities. There was neither skin rash nor joint effusions. Edema was graded at 1+ bilaterally in his lower extremities. Urine sediment showed microscopic hematuria, with some dysmorphic features. Serologic studies showed elevated Kappa/Lambda ratio at 2.52, but no paraprotein per serum and 24-hour urine protein electrophoresis. He had normal C3 & 4 levels, negative antinuclear and anti-neutrophil cytoplasmic antibodies, and his hepatitis B viral surface antigen and hepatitis C viral antibody were also unremarkable. Erythrocyte sedimentation rate was 37. His age-appropriate cancer screening was unrevealing. Kidney ultrasound showed normal sized kidneys with scattered cysts and normal appearing parenchyma.

**Figure 1:**
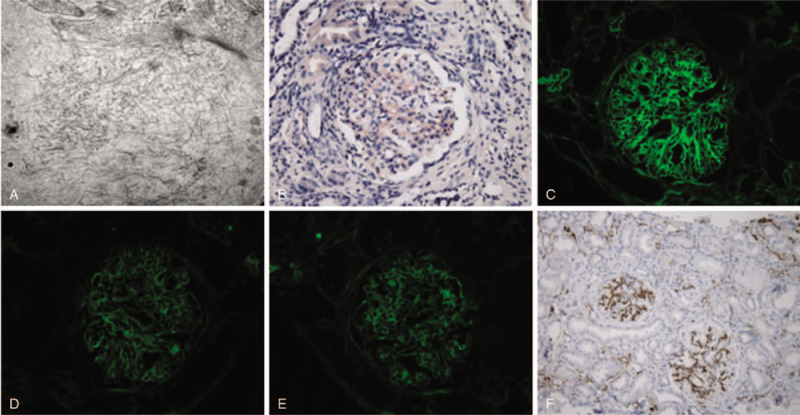
Images of kidney biopsy. A. Electron microscopy (80000X) showing fibril deposits; B. Congo red stain under light microscopy; C. Immunofluorescence stain for IgG (400X); D. E Immunofluorescence stain for Kappa and Lambda light chains respectively (400X); F. DNAJB9 stain under light microscopy.

The patient underwent renal biopsy, which showed mesangioproliferative glomerulonephritis, with both mesangial and subendothelial fibrillary deposits (Fig. 1, Image A). The fibrils measured 14.8 nm in diameter and were Congo Red positive (Fig. 1, Image B). Immunofluorescence were positive for polytypic IgG (predominant IgG4) (Fig. 1, Image C) and C3. Staining for serum amyloid A was negative. There were equal and trace reactivities of Kappa and Lambda light chain in the glomeruli and tissue background (Fig. 1, Image D& E). Lastly, there were advanced chronic changes noted in the parenchyma, including global and segmental glomerulosclerosis (52% of glomeruli) and significant interstitial fibrosis (60% of the cortex).

Considering atypical findings on renal biopsy including larger than expected fibril size of 14.8 nm (usually < 10 nm in amyloidosis), negative amyloid protein A staining, and lack of clinical evidence of extrarenal amyloidosis, we decided to send his specimen for a special DNAJB9 stain (Fig. 1, image F) which is a biomarker specific for FGN.^[5]^ In the meantime, he was started on MMF 1 gram twice daily and oral prednisone 40 mg daily (0.5 mg/kg/d) for a total of 6 months with a slow taper of prednisone afterwards in light of clinical factors favoring a diagnosis of FGN, despite the positive Congo Red stain. His serum creatinine trended down from 3.2 to 1.5 mg /dL in next 6 months, and his proteinuria improved to baseline with repeat random urine albumin-to-creatinine ratio reduced to 431 mg/g. His DNAJB9 stain later came back positive.

## 3 Discussion

FGN is a rare but well recognized pathological entity. It belongs to a group of glomerular diseases known as fibrillary glomerulopathies, which also include amyloidosis. FGN has the unique ultrastructural feature of haphazardly arranged, straight fibrils in the mesangium and/or along the glomerular basement membrane. The slightly larger fibrils and Congo Red negative deposits distinguish FGN from amyloidosis. This histological distinction using Congo Red is important clinically because while the treatment for amyloidosis often involves chemotherapy and autologous stem cell transplantation,^[6]^ the treatment for FGN relies on immunosuppression.^[7]^

However, rare congophilic FGN has been recently reported by a group of Mayo investigators and was observed in the case we presented. Alexander et al examined kidney biopsy specimens containing Congo Red–positive glomerular deposits from 2010 to 2017. Some were originally referred for amyloid typing. After comprehensive analysis, a total of 18 specimens were found to be unusual examples of FGN. They accounted for 4% of FGN in which Congo Red staining was performed. There was a male predominance, and a higher incidence of concomitant monoclonal gammopathy was observed. However, there appeared to be no differences in serum creatinine, and incidence of hematuria or nephrotic syndrome at presentation, when compared to traditional FGN. EM showed the typical randomly oriented nonbranching fibrils in the mesangium and glomerular basement membranes with fibril sizes ranging from 11 to 18 nm.^[8]^ The techniques used to confirm a diagnosis of FGN were liquid chromatography–assisted tandem mass spectrometry of microdissected tissue sections and DNAJB9 immunohistochemistry.

DNAJB9 is a protein involved in the stress response of endoplasmic reticulum and is found to be highly concentrated in the glomerular capillary and mesangium of patients with FGN. DNAJB9 staining has not been seen in other glomerular diseases including amyloidosis or in healthy subjects, and therefore is a marker for FGN with a reported 100% sensitivity and 100% specificity.^[5,9,10]^

Our patient is a unique case of an already rare congophilic FGN, which to our knowledge, is the first reported case of successful treatment of this rare condition. In general, renal survival in traditional FGN is poor, with over 40% of patients reached ESRD within 4.3 years according to a case series.^[4]^ Aside from kidney transplantation, there is currently no proven effective therapy for FGN. As in the traditional Condo Red-negative FGN, the response to treatment and renal prognosis also appears to be poor in congophilic FGN. Thus far, the only reported clinical data on these congophilic cases have come from the Mayo series. According to the authors, clinical follow-up was available in 16 out of 18 patients. Mean duration of follow-up was 23 (range, 4–56) months. Five (31%) progressed to ESRD and the remaining 11 (69%) had decreased kidney function, with persistent proteinuria and hematuria. No patient had normalization of serum creatinine level or > 50% decrease in proteinuria on follow up, except for one who with remission of proteinuria but worsening serum creatinine level.^[8]^ The immunosuppressive regimen included steroids alone, cyclophosphamide, azathioprine, rituximab alone or in combination with steroids.^[8]^ The fact that our patient showed impressive response to the treatment with MMF and prednisone is encouraging, and future larger scale studies would be needed to examine the efficacy of this treatment regimen.

## Author contributions

**Conceptualization:** Jie Tang.

**Data curation:** Pulkit Gandhi, Jie Tang.

**Investigation:** Jie Tang.

**Project administration:** Jie Tang.

**Resources:** Jie Tang.

**Supervision:** Jie Tang.

**Validation:** Jie Tang.

**Writing – original draft:** Pulkit Gandhi, Jie Tang.

**Writing – review & editing:** Jie Tang.
